# Cervical cord reperfusion injury: a rare complication of spine surgery

**DOI:** 10.1186/s12245-022-00443-3

**Published:** 2022-08-25

**Authors:** Wafa Hasan, Khalid Khan, Najla Alomani

**Affiliations:** Department of Radiology, Salmaniya Medical Complex, Manama, Bahrain

**Keywords:** Reperfusion injury, Cervical myelopathy, Magnetic resonance imaging, Case report, Cervical spine decompression

## Abstract

**Background:**

Anterior cervical discectomy and fusion procedure has been considered the surgical procedure of choice for the management of degenerative cervical myelopathy. Postoperative paresis is one of the most serious and concerning complications. The occurrence of such complications without any clear anatomic explanation (e.g., epidural hematoma) is unusual.

**Case presentation:**

A 62-year-old man underwent cervical spine decompression via the anterior approach for marked central canal stenosis and spinal cord compression due to degenerative changes. The operation was performed under neuromonitoring, and a complete discectomy for the levels C3/C4, C5/C6, and C6/C7 was performed. Fluoroscopy confirmed the correct placement of the inserted plates and screws. No motor or sensory deficits were evident after postoperative recovery. However, 1 day later, the patient experienced progressive weakness in his both upper and lower extremities. A whole spine MRI study was performed to exclude epidural hematoma as the possible etiology. Although no localized fluid collection or hematoma was observed, the study demonstrated abnormal signal intensity in the spinal cord on T2-weighted images at the levels C5 to C7. Such findings were consistent with changes in cord reperfusion syndrome. The patient was administered intravenous methylprednisolone therapy. Gradual improvement in the muscle power in his both extremities was noted, and the patient was discharged with a satisfactory outcome. One month later, the MRI study was repeated and showed regression of the previously seen high T2 signal intensity in the cervical spine.

**Conclusion:**

Cervical cord reperfusion injury is an extremely rare etiology of neurological deterioration following spinal cord decompressive surgeries. Clinicians need to maintain a high index of suspicion for this complication and should be familiar with its imaging appearance.

## Background

Degenerative cervical myelopathy is a common degenerative disease in the adult population, accounting for over 50% of non-traumatic injuries of the spinal cord in the USA [1]. It is associated with substantial disability and reduced quality of life [2]. The conservative measures have a limited role in the management of cervical myelopathy. Hence, the number of spine surgeries for degenerative cervical spine disease has been increasing. Anterior cervical discectomy and fusion procedure has been considered the surgical procedure of choice given its relative simplicity and safety [3]. The reported complication rate from anterior cervical discectomy and fusion varies in different studies but may reach up to 20% [4]. Such complications include dysphagia, recurrent laryngeal nerve injury, wound infection, esophageal perforation, radiculopathy, and instrument failure. Here, we report the case of a patient who developed cervical cord reperfusion injury following anterior cervical discectomy and fusion procedure, which is a rare but important complication.

## Case presentation

We present the case of a 62-year-old man who was brought to the emergency department because he was found to have difficulty in walking as he sustained multiple falls over the preceding 2 days. He did not experience any limb weakness or numbness. There was no history of an altered state of consciousness or slurred speech. The past medical history of the patient was remarkable for multiple medical comorbidities, including longstanding hypertension and diabetes mellitus with resultant chronic renal disease. He had not undergone any previous surgeries. The social and family history was non-contributory.

On examination, the vital signs revealed a normal temperature (36.7°C), tachycardia (105 bpm), elevated blood pressure (148/92 mmHg), and normal respiratory rate. The patient was not noted to have any facial asymmetry. He was not able to maintain his posture with a positive Romberg’s test. His gait was wide-based, and he was not able to tandem walk. The heel-to-shin test was impaired bilaterally. Sensory examination revealed loss of tactile, vibratory, temperature, and proprioceptive sensations below the spinal level of T4. Further, the Babinski sign was positive, and the ankle and knee reflexes were increased. Examination of cardiorespiratory system revealed normal findings. Initial laboratory investigations, including hematological and biochemical parameters, were non-contributory.

**Fig. 1:**
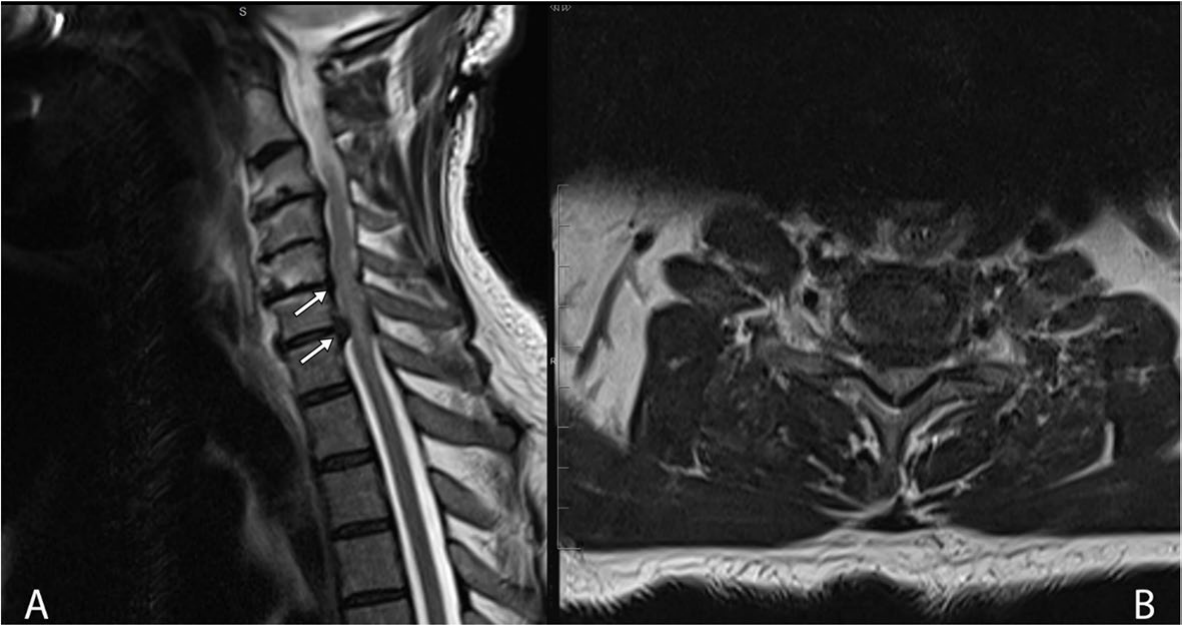
Pre-operative sagittal (**A**) and axial (**B**) T2-weighted MRI image of cervical spine demonstrated marked central canal stenosis at the C5/C6 and C6/C7 levels (arrows), resulting in spinal cord compression and myelopathic changes

The provisional diagnoses of the patient were transverse myelitis or central cord compression. The patient underwent magnetic resonance imaging (MRI) of the cervical spine, which demonstrated degenerative changes in the cervical spine, most notably at the C5/C6 and C6/C7 levels, with reduced intervertebral disc space, osteophyte formation, and posterior element degenerative changes in the form of facet joints arthropathy and thickening of ligamentum flavum resulting in marked central canal stenosis and spinal cord compression with increased T2 signal intensity at the affected levels representing myelopathy changes (Fig. 1).

The clinical and radiological findings were discussed in the multidisciplinary meeting. Surgical intervention for cervical spine decompression was planned. The procedure was made using the anterior approach. The surgery was performed under neuromonitoring. Complete discectomy for the levels C3/C4, C5/C6, and C6/C7 was performed. Integrated plate/cage devices placed after being pre-filled with bone graft. Then, screws have been inserted into each device. Fluoroscopy confirmed the correct placement of the plates and screws. The wound was irrigated and closed in a regular manner. The patient had uneventful recovery. Examination of the upper and lower limbs revealed no motor or sensory deficit postoperatively.

**Fig. 2:**
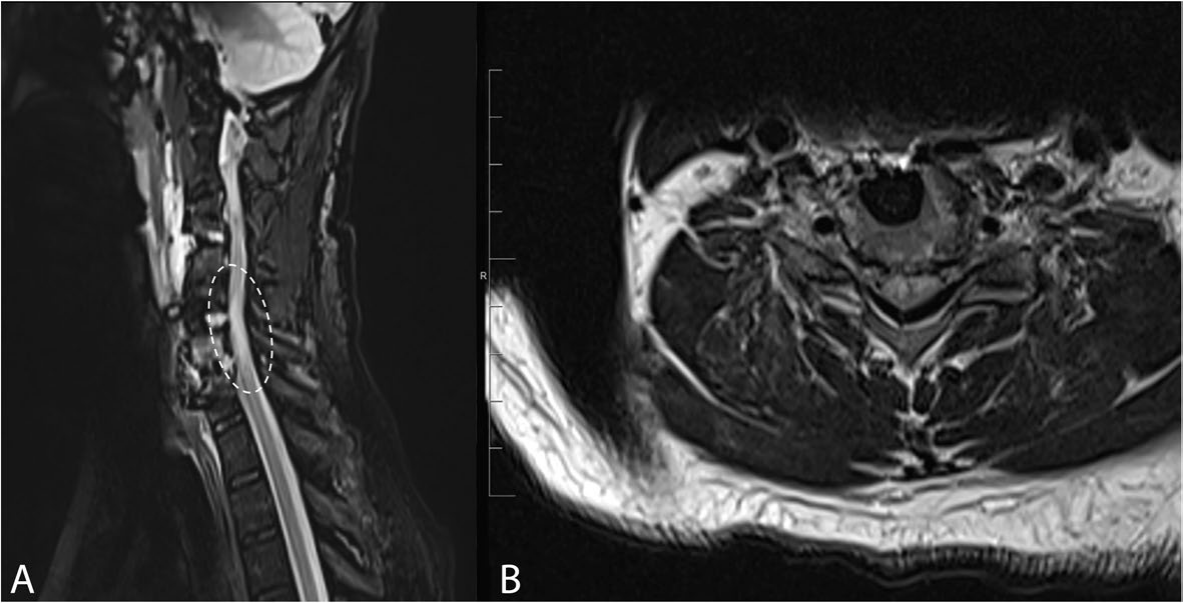
Post-operative sagittal STIR (**A**) and axial T2-weighted (**B**) MRI image showing cord swelling and abnormal high signal intensity at the levels C5 to C7 (encircled), representing changes of white cord syndrome

**Fig. 3:**
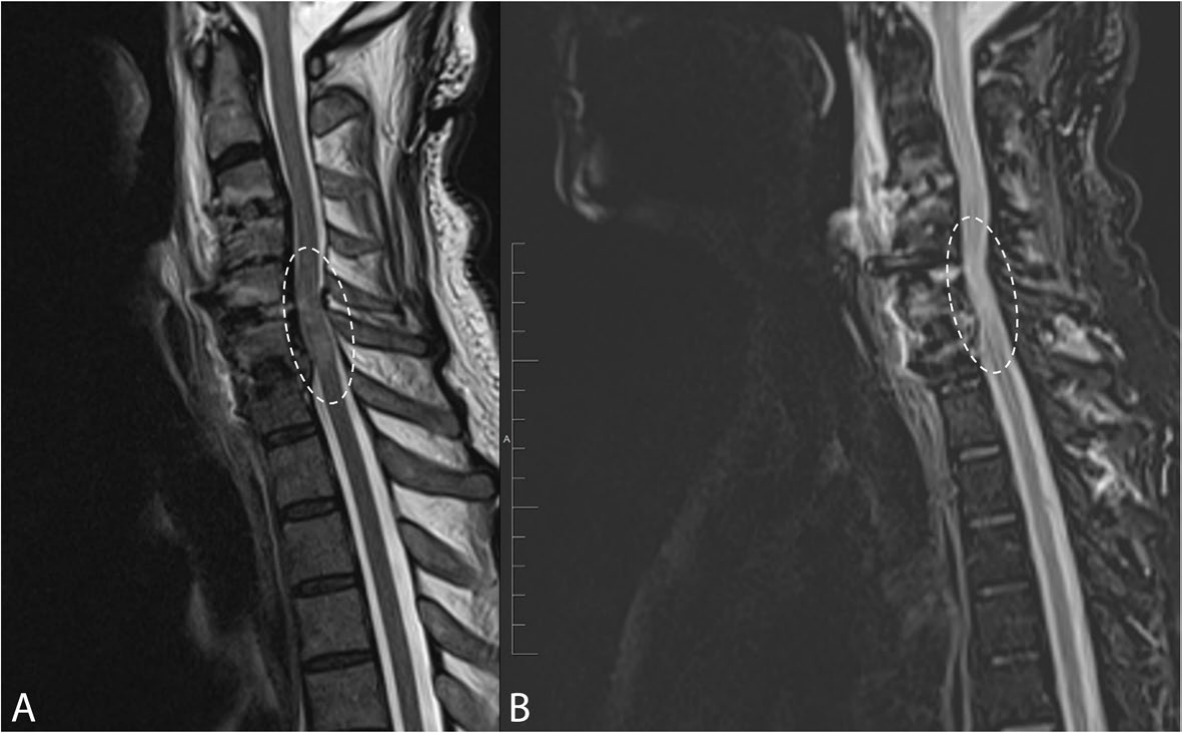
Post-operative sagittal T2 (**A**) and STIR (**B**) MRI images one month later showing regression of the previously seen high T2 signal intensity (encircled)

One day later, the patient experienced progressive weakness in his both upper and lower extremities with 0/5 motor strength throughout. Whole spine MRI study was performed to identify the underlying cause of neurological deterioration. The study demonstrated abnormal signal intensity in the spinal cord on T2-weighted images at the levels C5 to C7 (Fig. 2). However, no localized fluid collection or hematoma were observed. The aforementioned findings represented changes of cord reperfusion syndrome. The patient was administered intravenous methylprednisolone therapy at a dose of 8 mg twice daily for 4 days. Gradual improvement in the muscle power in his both extremities was noted. The patient was discharged in a satisfactory neurological outcome. One month later, the MRI study was repeated and showed regression of the previously seen high T2 signal intensity in the cervical spine (Fig. 3).

## Discussion

We reported the case of a cervical cord reperfusion injury, an extremely rare complication of anterior discectomy decompression and fusion procedure. This rare complication was first described by Chin et al. in 2013 [5] in a middle-aged man who underwent an elective anterior discectomy decompression and fusion and experienced incomplete tetraplegia postoperatively that was also noted during intraoperative somatosensory evoked potentials neuromonitoring. Postoperative paresis is one of the most serious and concerning complication after cervical spine surgery. Epidural hematoma is a typical etiology of postoperative paralysis, which can be detected by postoperative magnetic resonance imaging to allow for immediate revision to restore the spinal cord function [6]. Inadequate decompression, iatrogenic cord injury, intraoperative hypotension, and dislocated spinal implants are important causes to be considered in patients with postoperative neurological deficits after decompressive procedures [7, 8].

In the present case, the patient experienced sudden postoperative neurological deterioration and immediate magnetic resonance imaging was request for ruling out epidural hematoma. Considering that no clear anatomic explanation of the neurological deficit was evident, cervical cord reperfusion injury was the most probable etiology. This complication is often termed as a “white cord syndrome” because of its radiological appearance on magnetic resonance imaging with a high signal intensity of the spinal cord on T2-weighted images [5].

The pathophysiological of the cervical cord reperfusion injury can be explained by the increased blood flow after the sudden decompression of a possible ischemic segment of the spinal cord that leads to disruption of the blood-spinal cord barrier and release of oxygen-free radicals. It is assumed that the presence of myelopathy changes of the affected spinal cord on preoperative magnetic resonance images, as in the present case, may increase the tendency to develop the reperfusion injury [8].

Considering its rarity, no clear treatment guidelines have been established for the management of cervical cord reperfusion injury. However, the use of high-dose corticosteroid therapy with early physiotherapy interventions may result in neurological improvement. Further, keeping the mean blood pressure above 85 mmHg have been described [5, 8].

## Conclusion

Cervical cord reperfusion injury is an extremely rare etiology of neurological deterioration following spinal cord decompressive surgeries. Clinicians need to maintain a high index of suspicion for this complication and need to exclude other possible surgical complications. Early diagnosis and management with high-dose corticosteroid therapy are vital to restore the spinal cord function.

## Data Availability

Not applicable.
